# Tool Run-Out Measurement in Micro Milling

**DOI:** 10.3390/mi8070221

**Published:** 2017-07-24

**Authors:** Aldo Attanasio

**Affiliations:** Department of Mechanical and Industrial Engineering, University of Brescia, Via Branze 38, 25123 Brescia, Italy; aldo.attanasio@unibs.it; Tel.: +39-331-613-3181

**Keywords:** micro milling, tool run-out, experimental measuring, experimental tests, Ti6Al4V alloy, Industry 4.0

## Abstract

The interest in micro manufacturing processes is increasing because of the need for components characterized by small dimensions and micro features. As a result, researchers are studying the limitations and advantages of these processes. This paper deals with tool run-out measurement in micro milling. Among the effects of the scale reduction from macro to micro, tool run-out plays an important role, affecting cutting force, tool life, and the surface integrity of the produced part. The aim of this research is to develop an easy and reliable method to measure tool run-out in micro milling. This measuring strategy, from an Industry 4.0 perspective, can be integrated into an adaptive model for controlling cutting force, with the aim of improving the production quality and the process stability, while at the same time reducing tool wear and machining costs. The proposed procedure deduces tool run-out from the actual tool diameter, the channel width, and the cutting edge’s phase, which is estimated by analyzing the cutting force signal. In order to automate the cutting edge phase measurement, the suitability of two functions approximating the force signal was evaluated. The developed procedure was tested on data from experimental tests. A Ti6Al4V sample was machined using two coated micro end mill flutes made by SECO setting different run-out values. The results showed that the developed procedure can be used for tool run-out estimation.

## 1. Introduction

Micro manufacturing processes are becoming fundamental in several industrial fields. The need for having products characterized by very small dimensions and features is continuously increasing in biomedical, mechanical, automotive and aerospace applications. At the same time, many researchers are focusing their attention on manufacturing processes that are able to realize these micro components. As reported in [1], several different definitions of “micro machining” can be found in the literature. Dornfeld et al. [2] referred to their modified version of the Taniguchi graph [3], which shows the development of the manufacturing capability in terms of achievable machining accuracy, when they defined micro machining as a process in which the accuracy is lower than 1 microns. In Masuzawa et al. [4], Masuzawa et al. state that, although micromachining literally is a machining process with dimensions between 1 μm and 999 μm, it is possible to consider as micro machining processes all the processes utilized for realizing features that cannot be achieved by conventional ones. A similar definition is given in [5], where the production of micro parts is defined as “*the production of parts or structures with at least two dimensions in the sub-millimeter*”. Differently, Alting et al. [6] focused their attention on the definition of micro engineering as all the processes dealing with developing and manufacturing components with functional characteristics of at least one dimension of the order of millimeters. It is evident that all these definitions refer to different process issues, including feature dimension, process accuracy, surface roughness, feature feasibility, and so on. A definition covering all the issues characterizing a micro manufacturing process is hard or even impossible to give. For this reason, a general definition of micro machining has been assumed in this paper. This definition considers micromachining to be all the manufacturing processes that allow dimensions to tenths of a millimetre through using tools with sub-millimetre dimensions.

Of course, this definition covers a wide range of manufacturing processes. Micro cutting or micro mechanical machining processes are amongst them, and have many advantages. They are cost effective, flexible, efficient, and guarantee the possibility of realizing complex shapes with high removal rates, high accuracy, good roughness and low costs with respect to other non-conventional processes.

The study of micro cutting operations involves phenomena that can be neglected at the macro scale. These phenomena become important at the micro scale since the tool dimension, the uncut chip thickness and the material grain size are of the same order of magnitude. Consequently, a variation of characteristics from a general behaviour (i.e., size effect) can be observed when reducing the size of the workpiece, tool or process parameters [7]. Under these working conditions, one of the main aspects to be considered is the identification of the minimum chip thickness value under which the removal process is dominated by a ploughing regime instead of a shearing one. The ploughing regime is an undesired regime, since the material is not removed (i.e., the chip does not form); instead, it is just plastically deformed under the tool edge. It was demonstrated that the minimum chip thickness mainly depends on the tool material and its geometry [8], and the workpiece material and its microstructure [9]. The size effect also strongly affects the specific shear energy (SSE) needed for material removal. In fact, as reported in [8], SSE increases as the uncut chip thickness decreases.

When approaching micro milling processes [10,11,12], it is essential to understand how process parameters (feed per tooth, cutting velocity, and depth of cut), tool geometry (tool edge radius, helix angle, etc.) and the tool–machine pairs (tool–spindle run-out, machine stiffness, etc.) affect the process and the part quality. For this purpose, the development of analytical models suitable for describing cutting operations on a micro scale, is very helpful. Therefore, many papers in the literature are focused on modelling different issues of micro milling operations.

Yoon et al. [13] and Kim et al. [14] investigated the influence of tool deflection and the radial depth of cut on chip formation in micro milling. These works showed that the axial component of the cutting force can be neglected when the axial depth of the cut is limited to few microns. 

Garzòn et al. [15] analyzed the problems related to cutting force measurement during micro cutting operations. It was demonstrated that the dynamometers used in micro cutting operations must be characterized by high sensitivity and bandwidth because of the high spindle rotation regime. Zhu et al. [16] focused their attention on force signal and showed how to manage a micro milling force signal affected by strong noise. 

An analytical model for estimating unbalanced cutting forces due to tool run-out is proposed by Bissacco et al. [17]. In this model, different force profiles for each cutting edge are considered as a function of tool run-out.

In their model, Park et al. [18] considered the effects of tool deflection and tool run-out on the expression of tool trajectories. They suggested that the main phenomena characterizing the ploughing regime are ploughing and the elastic recovery of workpiece material, and that beyond a critical uncut chip thickness value, if elasto-plastic effects are negligible, then the cutting regime can be considered shearing dominant.

For studying tool deflection, Mamedov et al. [19] developed a Finite Element (FE) model including tool run-out, where the tool is considered as a cantilever beam with effective diameter in the fluted portion.

Bao et al. [20,21,22] developed an analytical model for micro-end milling operations, including tool run-out, while Afazov et al. [23] introduced a new approach for evaluating micro-milling cutting forces that considered ploughing regime and the effects of tool run-out.

An analytical model for predicting the three-dimensional cutting forces of micro-end milling processes, based on the trochoidal trajectory of the cutting edge and tool run-out, was developed by Li, et al. [24]. This model is based on six optimized coefficients, which minimizes the errors between the model and the experimental force values through the least square methods. They studied the influence of feed per tooth and tool run-out on the uncut chip thickness of each cutting edge. The limitation of this model is that the six coefficients are not constant; they are optimized at every spindle rotation. Consequently, this procedure requires high computational time. Attanasio et al. [25] proposed a procedure based on the particles swarm optimization strategy for calibrating the coefficients of an analytical model of cutting force in micro milling, including tool run-out.

Other works [26,27,28,29,30,31,32] dealing with the development of micro-milling cutting force models can be found in the literature, which demonstrates the high level of interest in this topic. All these papers agree on the need for these models to include the influence of cutting regimes (ploughing and shearing), the trajectory of the cutting edges, tool run-out, tool deflection, elastic recovery, and entry and exit angles. Amongst all these influencing factors, tool run-out plays an important role. This is because in micro milling, the ratio between tool run-out and feed per tooth is very high [32]. In some cases, tool run-out is so high that just one flute cuts the material, generating an asymmetric cutting [27].

The objective of this research is to develop an easy and reliable method to measure tool run-out in micro milling. The proposed strategy starts with realizing channels on a sample. During the process, the cutting force is acquired at a high sampling rate in order to have a high-resolution signal. After that, the channel width is measured with a microscope. A geometrical model then utilizes these data to provide the tool run-out value.

In order to study the limits of this method, a sensitivity analysis of the influence of the spindle speed on the result accuracy was performed, which showed the need to use a high-signal sampling rate. Moreover, with the aim of automating the proposed strategy, the suitability of two approximating functions, namely the Fourier series and the sum of sine, was estimated. The developed procedure was tested on data coming from experimental tests, which consisted of realizing microchannels with 800 μm width by using coated tungsten carbide micro mills on samples made of Ti6Al4V alloy.

## 2. Tool Run-Out in Micro Milling

Tool run-out is a phenomenon that occurs due to the sum of the geometrical displacements of the spindle axis, tool-holder axis and tool axis from the theoretical rotation axis. This sum produces a deviation between the theoretical cutting edge’s trajectory and the actual one. 

**Figure 1:**
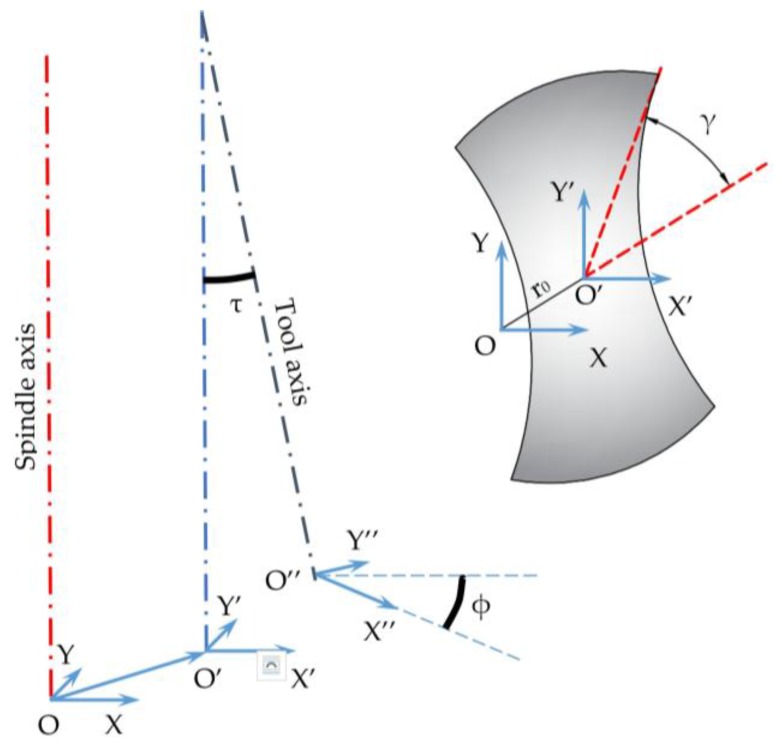
Tool run-out and its geometric parameters.

As described in [28], from a geometric point of view, tool run-out can be summarized by four parameters (see Figure 1). Two parameters define the axis offset, namely, offset distance or run-out length (r_0_) and offset or run-out angle (γ_0_); the other two parameters define the axis tilt, namely, tilt angle (τ) and the location angle of the tilt angle (ϕ).

Typically in conventional milling, since the mill diameter is very large compared to tool run-out, this error has a low influence on the cutting process. In micro milling, because of the limited tool dimension, the influence of tool run-out on the process cannot be neglected. In particular, tool run-out causes unbalanced chip thickness between the mill teeth. Consequently, different cutting forces arise on each cutting edge. This unbalanced load on the teeth generates unavoidable vibrations that affect the process stability. Therefore, minimizing tool run-out in micro milling is essential in order to increase the final quality of the surface finish, avoid accelerated tool wear or even tool breakage, and avoid the inception of undesired vibrations (i.e., increase the stability of the process).

However, the tool run-out parameters are not easy to measure in micro milling. In the literature, different approaches can be found. These strategies often measure tool run-out on the tool shank. However, as pointed out in Li et al. [32], tool run-out cannot be estimated at the tool shank in micro milling because of the additional run-out due to the tapered area of the tool that connects the cutting edges to the tool shank. In particular, dial indicators [23,27,28], microscopes [29], capacitive sensors [30], and laser sensors [31] have been utilized for this purpose. Other strategies defined the values of the run-out parameters by optimization algorithms [25,32,33]. As a general remark, when applying these methods, the cutting force signals coming from experimental data are utilized for setting the best run-out values that minimize an objective function. Applications of this strategy for tool run-out estimations can be found in conventional milling [34,35,36,37], which demonstrates there is also interest on tool run-out at the macro scale. All this research aims to provide a reliable tool that is able to estimate tool run-out during micro or macro milling. From an Industry 4.0 perspective, these tools can be integrated into an adaptive model designed for cutting force control [38], with the aim of improving the production quality and the process stability, while also reducing tool wear and machining costs.

## 3. Geometrical Model of Run-Out

**Figure 2:**
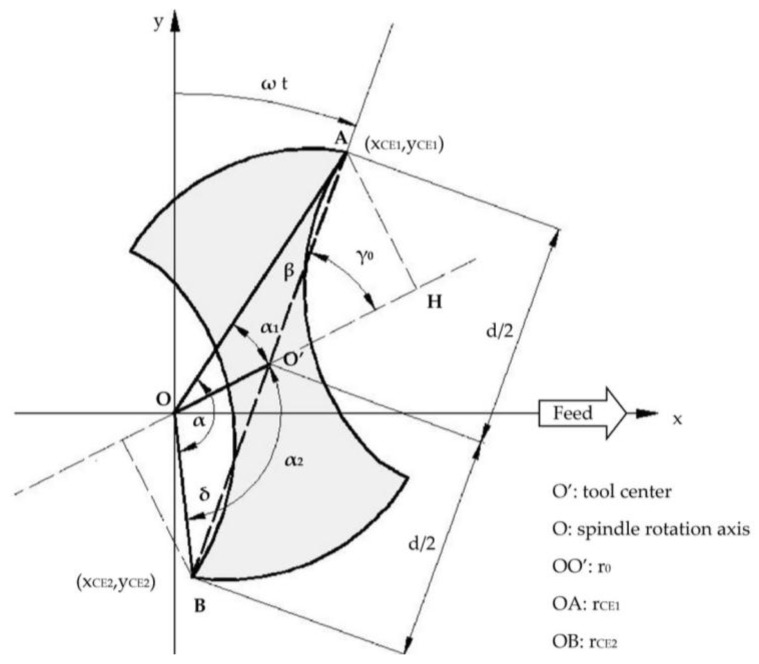
Micro end mill configuration with tool run-out.

The present geometrical model of tool run-out neglects tilt angle and the location angle of tilt angle (Figure 1). This choice was made after considering that when performing micro-machining operations, the use of suitable machine tools—namely nano-precision, ultra-precision or high-precision machine tools, depending on the accuracy level—is strongly suggested. These types of machine tools typically guarantee tilt angles lower than a hundredth of degree. It is evident that when using standard or in-house machine tools [28], this simplification is not acceptable. Figure 2 schematizes the considered configuration; run-out length (r_0_) and run-out angle (γ_0_) are reported.

In a previous work [25], the author presented a geometrical model of the cutting edge trajectories of a two-flute micro end mill that considered tool run-out.

Three effects are due to tool run-out:

1. The radius of the first cutting edge (r_CE1_ in Figure 2) increases according to Equation (1);
  (1) $$r_{\mathrm{CE}1}\;=\bar{\;\mathrm{OA}}\;=\;\left(\frac{d}{2}{\;+\;r}_{0}\right)\cdot \sqrt{1\;+\;\frac{d\cdot r_{0}\cdot \left(\cos\gamma \;-\;1\right)}{{\left(\frac{d}{2}{\;+\;r}_{0}\right)}^{2}}}$$
2. The radius of the second cutting edge (r_CE2_ in Figure 2) decreases according to Equation (2);
  (2) $$r_{\mathrm{CE}2}\;=\;\bar{\mathrm{OB}}\;=\;\left(\frac{d}{2}\;-r_{0}\right)\cdot \sqrt{1\;-\;\frac{d\cdot r_{0}\cdot \left(\cos\gamma \;-\;1\right)}{{\left(\frac{d}{2}\;-{\;r}_{0}\right)}^{2}}}$$
3. The phase between the first and second cutting edge (referred to as cutting edge phase in the following) changes from π to α.

Concerning the cutting edge phase (α), this parameter can be estimated as the sum (Equation (3)) between α_1_ and α_2_ angles (Figure 2), i.e., the angle between the radius of the first cutting edge and the run-out length, and the angle between the radius of the second cutting edge and the run-out length.

These angles can be derived by Equations (4) and (5).

(3) $$\alpha \;=\hat{\;\mathrm{AOB}}{\;=\;\alpha }_{1}{\;+\;\alpha }_{2}$$

where

(4) $$\alpha_{1}\;=\hat{{\;\mathrm{AOO}}^{\text{′}}}\;=\;\mathrm{arcos}\left[\frac{r_{0}+\;\frac{d}{2}\cdot \cos\gamma }{r_{\mathrm{CE}1}}\right]$$

(5) $$\alpha_{2}\;=\hat{{\;O}^{\text{′}}\mathrm{OB}\;}=\;\mathrm{arcos}\left[\frac{r_{0}\;-\;\frac{d}{2}\cdot \cos\gamma }{r_{\mathrm{CE}2}}\right]$$

As demonstrated in [25], the trajectories of the first cutting edge (A in Figure 2) and the second cutting edge (B in Figure 2) can be expressed by Equations (6)–(9).

(6) $$x_{\mathrm{CE}1}{\;=\;r}_{\mathrm{CE}1}\cdot \sin\left(\omega t\right)\;+\;\frac{f}{60}\cdot t$$

(7) $$y_{\mathrm{CE}1}{=\;r}_{\mathrm{CE}1}\cdot \cos\left(\omega t\right)$$

(8) $$x_{\mathrm{CE}2}{\;=\;r}_{\mathrm{CE}2}\cdot \sin\left(\omega t\;+\;\alpha \right)\;+\;\frac{f}{60}\cdot t$$

(9) $$y_{\mathrm{CE}2}{\;=\;r}_{\mathrm{CE}2}\cdot \cos\left(\omega t\;+\;\alpha \right)$$

where *f* is the tool feed defined by Equation (10).

(10) $$f=f_{z}\cdot z\cdot n$$

All these equations depend on tool run-out parameters (r_0_ and γ_0_); for this reason, their computation is fundamental.

In the following, it will be demonstrated how it is possible to estimate the values of tool run-out parameters through realizing microchannels and measuring the actual tool diameter (i.e., $\bar{\mathrm{AB}}$), the rotational radius of the first cutting edge (i.e., $r_{\mathrm{CE}1}\;=\;\bar{\mathrm{OA}}$), and the cutting edge phase (i.e., α).

Referring to the AOB triangle of Figure 2 and applying the sine law, it is possible to compute the δ and β angles (Equations (11) and (12)).

(11) $$\frac{\bar{\mathrm{AB}}}{\sin\alpha }\;=\;\frac{\bar{\mathrm{OA}}}{\sin\delta }\;\to \;\sin\delta \;=\;\frac{\bar{\mathrm{OA}}}{\bar{\mathrm{AB}}}\;\cdot \sin\alpha \;\to \;\delta \;=\;\mathrm{arc}\sin\left(\frac{\bar{\mathrm{OA}}}{\bar{\mathrm{AB}}}\;\cdot \sin\alpha \right)$$

(12) β=π-α-δ

Then, applying the law of cosines (Equation (13)), it is possible to calculate the rotational radius of the second cutting edge r_CE2_ ($\bar{\mathrm{OB}}$).

(13) $$r_{\mathrm{CE}2}\;=\;\bar{\mathrm{OB}}\;=\;\sqrt{{\bar{\mathrm{OA}}}^{2}\;+\;{\bar{\mathrm{AB}}}^{2}-\;2\cdot \bar{\mathrm{OA}}\cdot \bar{\mathrm{AB}}\cdot \cos\beta }$$

Now, referring to BOO′ triangle of Figure 2, the tool run-out length *r_0_* ($\bar{\mathrm{OO}\text{′}}$) can be obtained by utilizing the law of cosines (Equation (14)).

(14) $$r_{0}=\bar{\mathrm{OO}\text{′}}\;=\;\sqrt{{\bar{\mathrm{OB}}}^{2}+{\bar{O\text{′}B}}^{2}\;-\;2\cdot \bar{\mathrm{OB}}\cdot \bar{O\text{′}B}\cdot \cos\delta }$$

While the tool run-out angle ($\gamma_{0}$) is computed using the sine law (Equation (15)).

(15) $$\frac{\bar{\mathrm{OO}\text{′}}}{\sin\delta }\;=\;\frac{\bar{\mathrm{OB}}}{{\sin\gamma }_{0}}\;\to {\;\sin\gamma }_{0}\;=\;\frac{\bar{\mathrm{OB}}}{\bar{\mathrm{OO}\text{′}}}\;\cdot \sin\delta \;\to {\;\gamma }_{0}\;=\;\mathrm{arc}\sin\left(\frac{\bar{\mathrm{OB}}}{\bar{\mathrm{OO}\text{′}}}\;\cdot \sin\delta \right)$$

**Figure 3:**
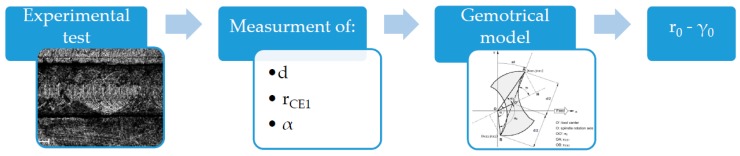
Procedure for computing tool run-out parameters (r_0_ and γ_0_).

The flow chart of Figure 3 summarizes the steps of the proposed procedure.

**Figure 4:**
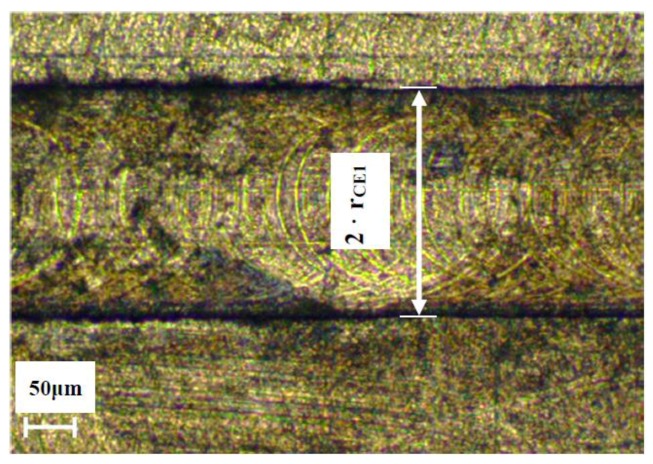
Experimental measurement of the rotational radius of the first cutting edge (r_CE1_) from channel width.

The measurement of the actual tool diameter (d) and the rotational radius of the first cutting edge (r_CE1_) are quite easy. The actual tool diameter can be directly measured from the tool. When realizing channels, the rotational radius of the first cutting edge can be measured from the channel width, which corresponds to two times the rotational radius of the first cutting edge, as shown in Figure 4. Several instruments are available to get these kinds of measurements, including: microscopes, coordinate vision-measuring machines, interferometers, capacitive sensors, and laser sensors.

However, measuring the cutting edge phase (α) is difficult. Microscopes can be used for this purpose [29], but this kind of approach cannot be used in the industry due to the high costs of the measuring system (microscopes, customized fixtures, etc.), the need of stopping the cutting operation whenever tool run-out needs to be measured, and, above all, the impossibility of automating this measurement.

**Figure 5:**
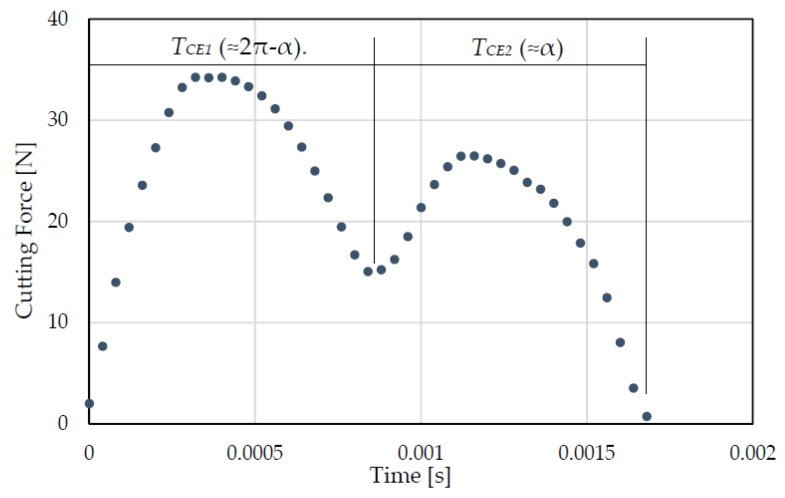
Experimental signal of cutting force for a two-flute micro end mill with run-out.

For these reasons, an indirect measuring of the cutting edge phase from the cutting force signal is proposed. The change of cutting edge phase from π to α causes a different cutting time between teeth. Referring to Figure 2, the cutting time of the first cutting edge (T_CE1_) is higher than that of the second cutting edge (T_CE2_). Figure 5 shows the typical curve representing the cutting force signal (one round) in milling when using a two-flute micro end mill in the presence of tool run-out. The different load and cutting time between the first and the second cutting edge are evident. Analyzing this signal, it is possible to measure the cutting time of each cutting edge.

The cutting time of each tooth depends on the cutting edge phase (α), and can be estimated by using Equations (16) and (17):

(16) $$T_{\mathrm{CE}1}\;=\;T\cdot \frac{2\pi \;-\;\alpha }{2\pi }$$

(17) $$T_{\mathrm{CE}2}\;=\;T\cdot \frac{\alpha }{2\pi }$$

where

(18) $${T\;=\;T}_{\mathrm{CE}1}{\;+\;T}_{\mathrm{CE}2}$$

is the cutting period.

Then, the cutting edge phase (*α*) can be derived by Equation (19).

(19) $$\alpha \;=\;2\pi \cdot \frac{T_{\mathrm{CE}2}}{T}$$

## 4. Experimental Set-Up

### 4.1. Measuring Systems and Sensitivity Analysis

Since the tool run-out parameters are indirectly measured, the accuracy of this measurement is relative to the accuracy of all the measuring instruments involved in the suggested procedure.

In this research, a confocal digital microscope (Hirox RH-2000) was used for measuring the actual tool diameter ($\bar{\mathrm{OA}}$) of the mill. This microscope guarantees a measuring accuracy of 0.8 μm. The channel width was measured using a fast-scanning autofocus 3D laser probe for surface texture measurement (Mitaka PF-60), characterized by an accuracy of 0.1 μm. As far as the cutting force is concerned, a Kistler load cell (code 9317C) was used. Three Kistler charge amplifiers (code 5015A) amplified the signals coming from the load cell, which gave three voltage output signals proportional to the cutting force components along the machine tool axis. Then, the amplified force signals were acquired by a National Instrument cDAQ-9174 equipped with a National Instruments 9205 board. This system guarantees an accuracy of the force measurement to 0.1 N.

It is important to highlight that, instead of the force measurement accuracy, the sampling rate of the cutting force signal (i.e., the interval time between two samples or the number of samples in a period) affects the measurement accuracy of the cutting edge phase (α). This is because the cutting edge phase (see Equation (19)) is a function of the ratio between cutting edge time and the cutting period. So, a higher accuracy can be obtained by setting a high sampling rate. The limit of the sampling rate depends on the acquisition system, but other aspects have to be considered when selecting the sampling rate. A high sampling rate generates large storing files (up to GBs); moreover, the higher the sampling rate, the higher the signal noise. In this research, the sampling rate was limited to 50 kHz; an acceptable file size (less than 100 Mb) and signal noise were obtained in this manner.

**Figure 6:**
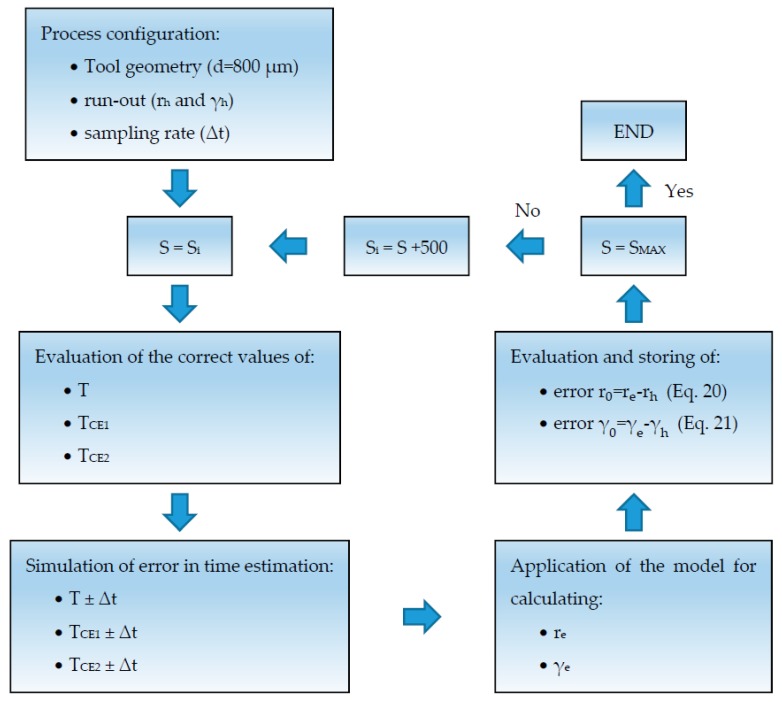
Flow chart of the sensitivity analysis of spindle speed influence on procedure accuracy.

Before realizing the cutting tests, an analysis of the influence of the cutting speed on the accuracy of the proposed procedure was done with the aim of defining the spindle speed to use during the experimental tests. Figure 6 schematizes how the sensitivity analysis was conducted.

The first step defines a process configuration comparable to the actual process. Thus, it was supposed to have a tool with a diameter of 800 μm characterized by a run-out length of three microns (r_h_), a run-out angle of 20 degrees (γ_h_), and a sampling rate of 50 kHz to acquire the cutting force. In this sampling rate, the interval time between two consecutive samples (Δt) is equal to 0.00002 s. Then, the analysis iteratively changes the spindle speed, increasing its value from 500 rounds per minute per iteration up to 50,000 rounds per minute, which corresponds to the maximum spindle speed of the machine tool used in this research. For each spindle speed (i.e., for each iteration), the correct cutting period (T) and the cutting time of each cutting edge (T_CE1_ and T_CE2_) are calculated. The following step adds and subtracts the Δt value to these time intervals, defining the maximum error achievable in time estimation. The geometrical model is then applied using these wrong times for calculating the run-out parameters (r_e_ and γ_e_). Finally, the maximum errors are estimated applying Equations (20) and (21).

(20) $${\mathrm{error}\;r}_{0}{\;=\;r}_{e}\;-{\;r}_{h}$$

(21) $${\mathrm{error}\;\gamma }_{0}{\;=\;\gamma }_{e}\;-{\;\gamma }_{h}$$

**Figure 7:**
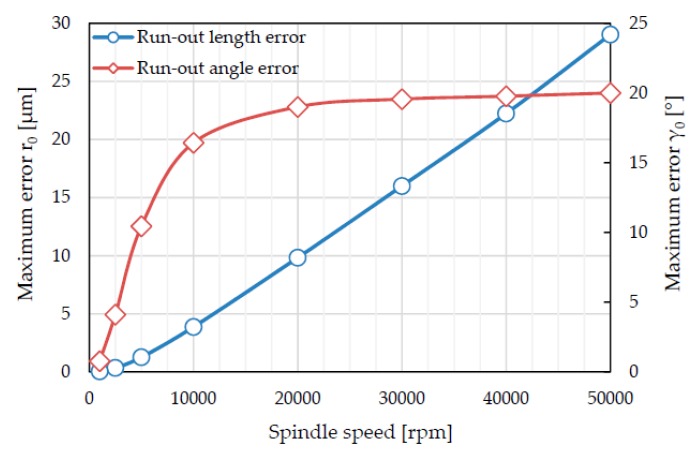
Errors in estimating the tool run-out parameters as a function of the spindle speed (d = 800 μm; r_0_ = 3 μm; γ_0_ = 20°; sampling rate = 50 kHz).

The results of the sensitivity analysis are reported in Figure 7. The maximum measuring errors of tool run-out length and angle decrease as the spindle speed decreases. In particular, the run-out length error shows a linear trend; while the run-out angle error is quite constant for spindle speeds higher than 10,000 rpm, it shows a rapid drop for spindle speed lower than 10,000 rpm.

In conclusion, the sensitivity analysis suggests that spindle speeds lower than 5000 rpm guarantee the lower run-out lengths and angle errors.

Taking these results into account, the spindle speed was set at 4166 rpm. In this manner, 720 samples per period (i.e., a sample every 0.5 degree of tool rotation) were acquired. This choice limits the maximum error of run-out length and run-out angle parameters. Because of the small tool dimensions, this value of spindle speed gives a low cutting speed (10.5 m/min). However, this cutting speed is acceptable for cutting Ti6Al4V alloy.

### 4.2. Experimental Test

**Figure 8:**
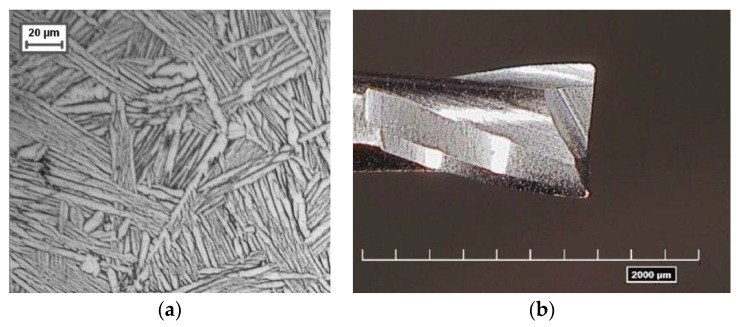
(**a**) Widmanstätten microstructure of Ti6Al4V sample; (**b**) Micro end mill (SECO-JM905L008-MEGA-T).

The experimental tests consisted of milling under the same cutting conditions: micro channels on a Ti6Al4V alloy sample. The sample material was heat treated [11,12] in order to obtain a fully or lamellar microstructure. Figure 8a shows a micrograph of the sample after 20 s of etching with Kroll. Table 1 and Table 2 summarize the chemical composition and the mechanical properties of the sample material, respectively.

The tests were performed on a five-axis Nano Precision Machining Centre KERN Pyramid Nano equipped with a Heidenhain iTCN 530 numeric control. A coated two-flute tungsten carbide micro end mill (Figure 8b) with a 800 μm diameter made by SECO (specification SECO-JM905L008-MEGA-T) was used. The PVD coating was nitride of titanium and aluminium, a suitable material for cutting titanium alloys. A confocal digital microscope (Hirox RH-2000) was used to measure the cutting edge radius, obtaining a value of 4 μm.

The realized channels consisted of rectangular slots with a depth of 100 μm, a width equal to the tool diameter, and a length of 12 mm.

Table 3 summarizes the cutting parameters utilized during the tests. According to the tool data sheet, a feed per tooth higher than the cutting edge radius was selected to avoid ploughing regime.

**Figure 9:**
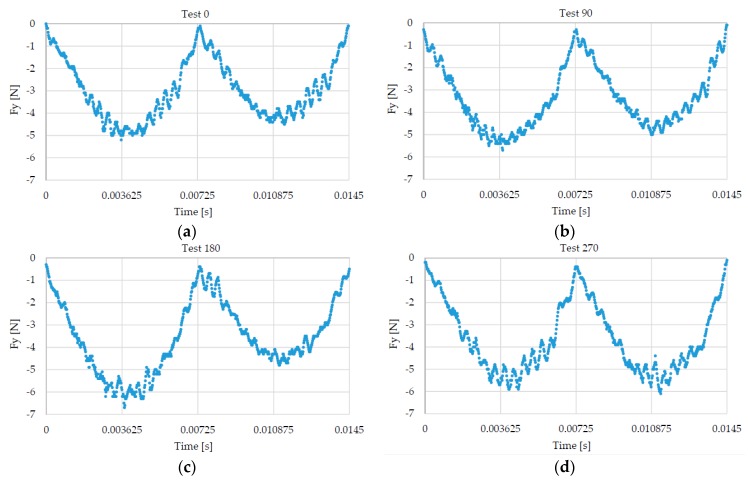
Cutting force component along *Y*-axis. One round. (**a**) Test 0; (**b**) Test 90; (**c**) Test 180; (**d**) Test 270.

An easy strategy was used to have different run-out conditions under the same cutting conditions. The same tool was used for realizing all the tests. At the end of each cut, the tool holder was unmounted from the spindle to check the tool wear and ensure that the tool wear did not affect the cutting force signal. Due to the low cutting length, no evidence of tool wear was highlighted during these control phases. In particular, the cutting edge radius was monitored to guarantee the shear-cutting regime during the experiments. In fact, an excessive increase of the cutting edge radius can change the cutting regime from shearing to ploughing. The conducted measures confirmed that the cutting edge radius was constant (4 μm) during all the tests. Then, the tool holder, rotated ninety degrees, was mounted on the spindle and a new channel was produced. In this manner, the tool run-out randomly changes for each test. Four channels were made applying this procedure, obtaining four different run-out conditions (i.e., Test 0, Test 90, Test 180, and Test 270). Figure 9 shows the cutting force signal measured along the *Y*-axis for each test; the higher tool run-out is noticeable for Test 180 (Figure 9c), the lower for Test 270 (Figure 9d).

### 4.3. Fitting Functions Analysis

In order to automate the procedure for the calculation of the cutting edge phase (α), different functions fitting the cutting force signals were tested. Matlab^®^ environment was used for this purpose. Since the force signal is used to measure the cutting edge’s time and the cutting periods, the fitting function analysis was applied to the force component signal along the *Y*-axis. The same results can be indifferently obtained considering the force component along *X* or *Z* directions.

**Figure 10:**
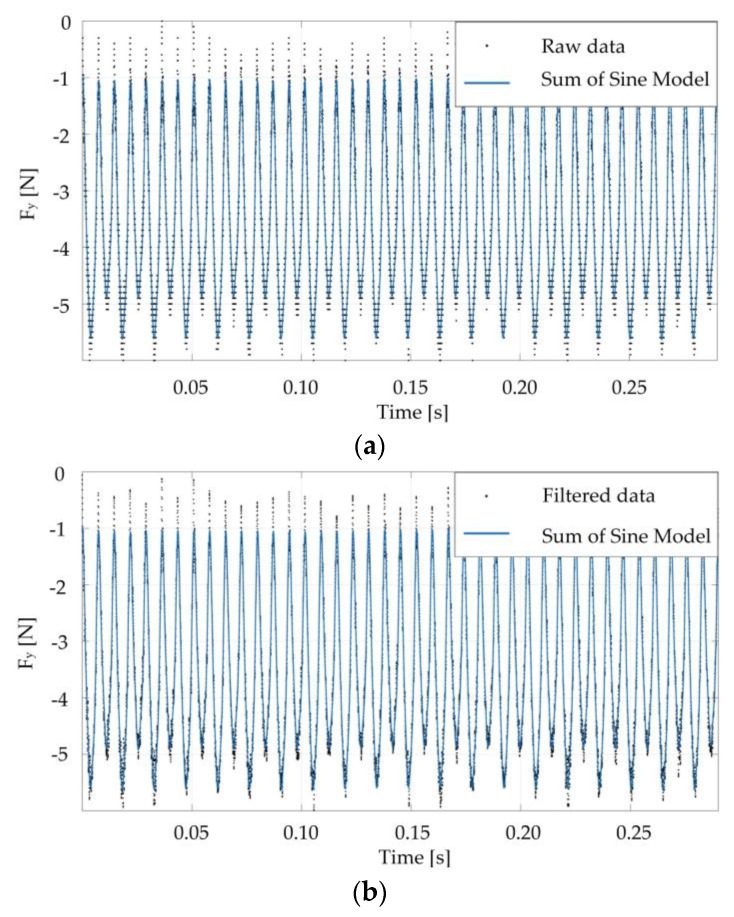
Sum of sine fitting function obtained from rough (**a**) and filtered (**b**) experimental cutting force components along the *Y*-axis (Test 90).

In particular, for each test, a portion of the signal of the experimental cutting force corresponding to twenty rounds was extracted. Then, the coefficients of two fitting functions, namely Fourier series and the sum of sine model, both limited to eight components, were calculated through the curve-fitting application of Matlab^®^. The same analysis was repeated on data filtered using Central Moving Average (CMA) filter, with a subset size equal to five. In this manner, the influence of the noise of the cutting force signal on the estimation of the cutting edge’s time and cutting period should be reduced. The graphs of Figure 10 report the superimposition between experimental data (raw and filtered) and the sum of sine model fitting for Test 90.

Once the fitting curves coefficients were defined, a Matlab^®^ script allowed the calculation of the cutting time of the first cutting edge (T_CE1_), the cutting time of the second cutting edge (T_CE2_), the cutting period (T), and, finally, the cutting edge phase (α).

## 5. Results and Discussion

**Figure 11:**
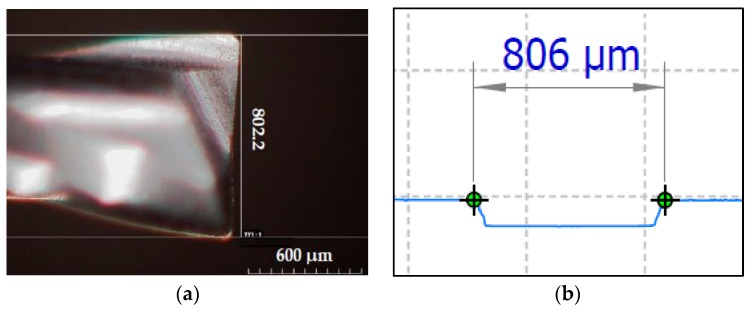
(**a**) Tool diameter measuring (Hirox RH-2000); (**b**) microchannel profile (Mitaka PF-60).

As mentioned previously, the tool diameter was measured by using a Hirox RH-2000 confocal microscope (Figure 11a); while the width of the four channels was estimated as an average value of measurements, using a Mitaka PF-60 laser probe in four different positions along the channels’ length. An example of output of the microchannel profile given by the Mitaka PF-60 measuring system is shown in Figure 11b. Table 4 summarizes the measuring results.

Table 5 reports the R-square goodness-of-fit statistic, the cutting time of the first cutting edge (T_CE1_), the cutting time of the second cutting edge (T_CE2_), the cutting period (T), and the cutting edge phase (α) obtained from the different fitting functions.

The high value of the R-square goodness-of-fit statistics confirms the suitability of all the selected fitting functions for correctly representing the cutting force signal. It is possible to use either the Fourier series or the sum of sine model indifferently, and to process raw or filtered data. The Fourier series applied to raw data is the preferred solution, due to the lower computational time.

In Table 5, the tool run-out parameter values (r_0_ and γ_0_) that were obtained applying the proposed procedure are also reported. As a general remark, it is possible to state that for each test, the fitting functions give very close run-out parameters. In agreement with the measured cutting force signals (see Figure 9), the higher run-out parameters were calculated for Test 180, and the lower for Test 270.

## 6. Conclusions

The purpose of this activity has been to design and develop a procedure that was able to measure the tool run-out length and angle parameters, and thus overcome the limits of the actual measuring strategies based on the use of microscopes, laser or interferometers. A geometric model involving the tool diameter, the channel width and the cutting edge phase, was introduced. For the automatic estimation of the cutting edge phase, the cutting edge force signal was processed through a procedure based on the curve-fitting application of Matlab^®^. Two fitting functions were tested by processing raw and data filtered through the Central Moving Average filter. The limitations of this procedure relate to the accuracy of the measuring systems used for estimating the tool diameter and the channel width. For this reason, measuring instruments with high accuracy were used. The results obtained applying this procedure on experimental data demonstrated the possibility of calculating the run-out parameters with very good accuracy. 

From an Industry 4.0 perspective, this procedure can be easily implemented in a cyber–physical system that, through changing the cutting parameters as a function of the measured tool run-out, will improve the quality of the micro-machined surfaces (i.e., surface roughness, geometrical accuracy, and surface integrity) and reduce the process costs. For this reason, further research will be focused on studying the influence of new materials, new tools dimensions and geometries, and new process parameters on this procedure. Moreover, research aimed to improve the accuracy of the measuring chain will be performed, which will make it possible to test higher cutting speeds.

## Figures and Tables

**Table 1:** Chemical composition of Ti6Al4V (percentage in weight).

Ti	Al	V	Fe	O
90%	6%	4%	<0.25%	<0.20%

**Table 2:** Mechanical and physical properties of Ti6Al4V.

Parameter	Value
Young’s Modulus	113.8 GPa
Yield Stress	880 N/mm^2^
Tensile Stress	950 N/mm^2^
Elongation at break	14%
Reduction at break	36%
Hardness	36 HRC
Notch impact	17 J
Poisson’s Coefficient	0.342
Density	4.430 kg/dm^3^
Thermal Expansion	8.6 μm/m·K
Thermal conductivity	6.7 W/m·K

**Table 3:** Cutting condition utilized during the experimental tests.

Parameter	Value
Cutting speed	10.5 m/min
Spindle speed	4166 rpm
Feed per tooth (*f_z_*)	10 μm/tooth
Axial depth of cut (*a_p_*)	100 μm
Radial depth of cut (*a_e_*)	Tool diameter
Lubrication	Dry

**Table 4:** Measured tool diameter and width of channels.

Parameter	Value
Tool diameter	802.2 μm
Channels width	
Test 0	807.2
Test 90	806.2
Test 180	806.5
Test 260	805.5

**Table 5:** Fitting functions results: T_CE1_, T_CE2_, T, α, r_0_, γ_0_, SSE and R^2^.

Test 0	Raw Data		Filtered Data	
Parameter	Fourier Series	Sum of Sine	Fourier Series	Sum of Sine
R-square	0.9645	0.9502	0.9708	0.9565
T_CE1_ [s]	0.007347	0.007371	0.007343	0.007373
T_CE2_ [s]	0.007153	0.007129	0.007157	0.007127
T [s]	0.01450	0.01450	0.01450	0.01450
α [°]	182.4	183.0	182.3	183.0
r_0_ [μm]	8.77	10.78	8.43	10.95
γ_0_ [°]	−74.0	−77.3	−73.3	−77.6
**Test 90**	**Raw Data**		**Filtered Data**	
**Parameter**	**Fourier Series**	**Sum of Sine**	**Fourier Series**	**Sum of Sine**
R-square	0.9781	0.9732	0.9825	0.9776
T_CE1_ [s]	0.007334	0.007300	0.007295	0.007300
T_CE2_ [s]	0.007166	0.007200	0.007205	0.007200
T [s]	0.01450	0.01450	0.01450	0.01450
α [°]	182.1	181.2	181.1	181.2
r_0_ [μm]	7.55	4.77	4.38	4.77
γ_0_ [°]	−75.2	−65.5	−63.1	−65.5
**Test 180**	**Raw Data**		**Filtered Data**	
**Parameter**	**Fourier Series**	**Sum of Sines**	**Fourier Series**	**Sum of Sines**
R-square	0.9740	0.9628	0.9787	0.9674
T_CE1_ [s]	0.007541	0.007531	0.007546	0.007531
T_CE2_ [s]	0.006959	0.006969	0.006954	0.006969
T [s]	0.01450	0.01450	0.01450	0.01450
α [°]	187.2	186.9	187.3	186.9
r_0_ [μm]	25.36	24.49	25.79	24.49
γ_0_ [°]	−86.9	−86.7	−87.0	−86.7
**Test 270**	**Raw Data**		**Filtered Data**	
**Parameter**	**Fourier Series**	**Sum of Sines**	**Fourier Series**	**Sum of Sines**
R-square	0.9737	0.9554	0.9780	0.9708
T_CE1_ [s]	0.007308	0.007252	0.007266	0.007256
T_CE2_ [s]	0.007192	0.007248	0.007234	0.007244
T [s]	0.01450	0.01450	0.01450	0.01450
α [°]	181.4	180.1	180.4	180.2
r_0_ [μm]	5.29	1.66	2.16	1.73
γ_0_ [°]	−72.2	−6.0	−40.2	−18.0
